# Statement of Twenty-Three Cases of Midwifery in Which Chloroform Was Administered

**Published:** 1849-06

**Authors:** Geo. N. Burwell

**Affiliations:** Buffalo


ART. 3.—*Statement of twenty-three cases of Midwifery in which Chloroform was administered.* By GEO. N. BURWELL, M. D.

In the number of this Journal for November last, I gave a statement of fifteen cases of Midwifery in which I had given Chloroform. Since that article was written (October 1st, 1848,) I have given it in twenty-three other cases, which I now submit to the readers of this Journal:—

*Case 1st.*—Mrs. McC. This was a stout hearty woman, in labor, Oct. 7th, with her fourth child. She had been in hard pain for two hours before I saw her. I found the os-uteri dilatable, yet it did not become fully dilated during the pains, but lay before the bag of waters somewhat thick, soft, and flabby, pulse 64; general condition good. I ruptured the membranes and gave Chloroform. The pains were instantly quickened. The Chloroform "softened" them, as she termed it, and made her feel as though she was going to sleep, but I did not get an opportunity to give it many minutes. The head soon escaped through the os-uteri and began to distend the perineum, and from this moment there was no cessation of pain until the child was born, scarcely enough to allow her to take breath. Her face was quite blue, so intensely congested did it become. Five minutes after the birth of the child her pulse was 100, and almost intermittent once in ten or fifteen beats; in ten minutes more it had fallen to 80 and was regular. The congestion of the face did not disappear for over half an hour.

The relief from the Chloroform was not so great as I had anticipated. The pains were too severe and rapid to be lessened by doses short of what I call full ones, that is drachm doses; while I gave but thirty or forty drops, and had that opportunity only for three pains. These then became so continuous, and accompanied with such congestion of the face (and of course of the brain,) I did not deem it prudent to urge it further. I would invite similar caution in similar cases. Chloroform ought not to be given when the patient is struggling for breath from severity of pain.

*Case 2nd.*—Mrs. D. Oct. 8th. A moderately stout woman, in her first labor. General health had been good. I first saw her at 10¼ A. M. The os-uteri dilated to about 1½ inches in diameter, thin, but not distensible, and not much influenced by the pains; membranes entire; the head presenting in the 1st position of the vertex. She had been in pain for thirty-six hours; especially so, for the last twelve hours. The intervals between the pains were about five minutes. Pulse 96, active—v. s. ad ℥xx.

I left to return at 11 o'clock—learned that the pains had been weak and inefficient, although at short intervals. On examination found no discernible progress of dilatation. At 11½ o'clock there had been some slight progress. I now made pressure upon the os-uteri during pains, by catching the anterior lip between the pulp of my finger and the pubes, and during pain gently carrying my finger from side to side. I felt surprised after three or four pains to find dilatation going on so much more rapidly. In fifteen minutes the waters escaped, and in five more the head passed through the os-uteri and reached the perineum. It was here delayed nearly half an hour.

I administered the Chloroform in this case a little over half an hour, commencing two or three pains before the waters escaped. From the first it had the effect of lessening her suffering, and towards the termination of the labor, she had got enough under its influence to be entirely unconscious of the birth of the child. This degree of anæsthesia was reached without even putting on the sponge more than twenty or thirty drops at one time, and without once continuing the inhalations in the intervals of pain.

With one or two exceptions towards the last of the labor, she was sensible enough to tell us of the accession of each pain, when the Chloroform was given; and it was taken away as soon as I saw she was eased by it. Twice during this time she became slightly dilerious after making eight or ten inspirations from the sponge freshly wet. The delirium was of an unpleasant nature, as, with a single exception, I have always noticed it to be, when produced by Chloroform. I never urge its use after this symptom shews itself, for I have always found it accompanied with insensibility to pain, the sole object sought for in the use of the remedy.

To the last she shewed a great desire for the Chloroform, making considerable exertion to breathe freely of it. She did not remain insensible over a minute or two after the birth of the child.

The placenta was thrown off in about fifteen minutes afterwards. My attention at this time was particularly attracted to what seemed to me to be very painful contractions of the womb; she groaned aloud with them. She said they were low down, below the pubes, not above; nor were they accompanied with that "rising" of the womb, so usual in after-pains. During the application of the binder, she desired to be pinned very tight, unusually so, I thought. The fourth of a grain of morphine was given for the pain, and its repetition directed if necessary. Pulse 92, skin moist, and face pale when I left, three-fourths of an hour after the birth of the child.

The blood I had taken was buffy enough to have come from a pneumonic patient. It had one curious appearance. Small cottony masses of lymph were attached to the edges of the buff, around its whole circumference, and were lying floating in the serum between the clot, and the sides of the bowl.

6 P. M. Found her free from pain; has been asleep a considerable portion of the afternoon; talks deliriously in her sleep; when awake appears well enough; eye and countenance firm; pulse risen to 120.

*Oct 9th.* Had a comfortable night; has not had any headache nor dizziness; skin moist, and of a comfortable heat; has neither pain nor soreness of the bowels or groins on pressure; pulse still 120; tongue heavily covered with whitish fur; no particular thirst; some oatmeal gruel relished very well this morning.

℞ Hydrarg. c. mit—Pulv. Doveri aa, gr. ij. *m.* ft. pulv. Give one every four hours, and follow in half an hour by a cup of light warm tea.

℞ Sodae carb. ℈i. Potass. nit. gr. x *m.*—To be dissolved in a tumblerful of water for a drink.

10*th.* Her bowels moved three times during yesterday afternoon and evening, with a good deal of pain, through the pelvis, although the discharges were liquid. Rested well yesterday, although not so well last night; has some soreness on pressure over the hypogastric region, and some in the right leg; lochia offensive; pulse 120; skin rather warm, but moist; more thirst than yesterday; tongue as before.

℞. Pulv. Rhei. gr. vi.—to be given at 9 o'clock—a like powder to be given at 1 o'clock, if the first does not operate; and if necessary ℥ss of castor oil at 5 o'clock.

*Evening.* No movement of the bowels; feels relieved since morning; can move better; was lying comfortably on her left side when I entered the room; pulse 120: heat of skin; repeat the oil at 10 o'clock.

11*th.* Her bowels moved three times about 3 o'clock this morning, and with some pain; experiences but little soreness on pressure over the bowels; no bloating; lochia scanty; urine natural quantity, and passed wtthout pain; but yields a heavy red sediment on cooling. She turns and moves about in bed easily, and without pain. Her skin keeps too warm, but is not hot or burning to the touch; does not sweat; breathing moderately hurried and short; lips red; cheeks quite pale; pulse 128; milk distending the breasts, but does not run from them easily.

℞. Hydrarg. c. mit. gr. j.; Pulv. Doveri. gr .ij *m.* ft. pulv.—Given every two hours; the soda and nitre as before.

*Evening.* No improvement of symptoms; has had some cough to-day, with uneasy sensations in the left side, at the edge of the ribs; pulse rising some, being full 130 to 140; thirst; milk unusually free for one in her condition, having sufficient for the child; no improvement of the tongue. V. S. ad ℥ x iv.—This blood buffy, but not to the extent of that drawn on the first day.

*Oct.* 12*th.* No particular change of symptoms, except in the seat of pain, that, this morning, being mostly in the left groin; does not suffer much from it except on pressure. Pulse full, 130. The thirst, heat of skin, and breathing as on yesterday morning. I determined to take 8 oz. more of blood: *first,* because there was an activity and wiriness of the pulse, although so rapid; *second,* because of the continued buffiness of the blood; and *third,* because of the continued tenderness of the left groin. The blood was also moderately buffy, but not to the extent to encourage farther depletion.

The use of calomel was discontinued. A blister was applied at night over the tender groin.

*October* 13*th.* Feels relieved; attributes much of it to the blister, which drew well. Pulse still 130; has less thirst, and less heat of skin; milk more free; lochia nearly stopped; bowels regular, and the discharge quite natural; Urine still high coloured.

Prescribed the soda and nitre drink, to be taken freely. Allowed a little lemonade; dov. powder at night.

*Oct.* 14*th* to 19*th.* Symptoms continued to improve slowly until the 19th, when I considered her as fully convalescent. The tongue and pulse were among the last symptoms to improve. Before the former had become even tolerably clean, the latter had fallen below 100; her fever had gone; appetite become good; and the urine nearly healthy.

She improved rapidly after the 19th, and got as stout and hearty as though she had not suffered a fit of sickness.

This is the only case of puerperal fever that has occurred to me after the use of chloroform; I have always looked upon it as a case of puerperal intestinal fever, with a strong tendency to inflammatory complication. I have never thought it was caused by the chloroform, but do not wish, myself, to decide the question. Each one is at liberty to draw his own conclusions; the case is faithfuly reported to him.

Had it been other than a puerperal patient, the disease would, by many, be called a case of inflammatory fever. The points of most interest are, —1st, the sudden and unusual pain experienced on the separation and expulsion of the after-birth—2d, the rapid rise of the pulse until it reached nearly to 140—3d, the inflammatory nature of the blood drawn—4th, the coming of the milk at the regular time, and its continuing so free, notwithstanding the amount of fever, the rapid pulse, and the depletion—the want of prominent local symptoms; that tenderness of the left groin was the only one that was fixed enough to require any special applications.

*Case* 3—Mrs. D.—*Oct.* 14*th*—A stout hearty woman in labor with her fifth child. I gave chloroform only during the last half hour, after a rupture of the membranes. It made her quite delirious two or three times, but, as usual, afforded such relief that she called for more. This delirium was only momentary; she was quite sensible of the birth of the child. Both did well; not the least sickness afterwards.

*Case* 4—Mrs. W.—17*th*—This was very similar to the third one, except there was no delirium produced. It was her third labour. She had had an epileptic fit when in labor with her second child, and feared one very much, this time. The chloroform did not cause the least uncomfortable feeling about the head. She breathed it very steadily during the last half hour, and was delighted with the relief obtained, although not once made insensible.

*Case* 5*th*.—Mrs. T., *Nov.* 17*th*. This was a young woman of quite a delicate constitution, in labor with her first child. I was called early in the evening. She had been in pain all the afternoon. The pains came on once in five or six minutes, but were inefficient. Os-uteri dilated to about one and a half inches in diameter; edges thin. At 12 o'clock (midnight) I commenced the administration of the chloroform, the pains getting, by this time, to be quite severe, although the dilatation was making slow progress.

She breathed it well, and was relieved by it; no unpleasant symptoms were produced, and I therefore continued it the most of the time, until a quarter to 3 o'clock A. M., when the child was born. She only occasionally slept under it; she seemed disposed to talk; once she exclaimed that it made her crazy; she would frequently ask for it, and be apparently sensible when she had taken enough of it, and would then push the sponge from her mouth. Some five or six drachms were used. She had no headache, nor did it excite cough. Her recovery was rapid and perfect. The child did well.

*Case* 6*th*.—Mrs. H., *Dec.* 4*th*. This patient asked for it, knowing that I sometimes gave it. It was her second labor. It was rapid, and she only had an opportunity of breathing it about twenty minutes. She got enough, however, so as to be scarcely sensible of the birth of the child. She did well.

*Case* 7*th*., *Dec.* 18*th*. I first saw this patient at 3 o'clock in the morning. The women in attendance, expressed their gratification at seeing me, fearing I would be too late. Her pains were, indeed, so sharp and rapid, that I at first thought she would soon be through. But on examination, I found the os-uteri so closed, as scarcely to admit the end of my finger. It was her first labor. Her age, about twenty-five, I should judge. She had suffered severely from vomiting and sour stomach, and sore mouth, a great deal of the time during pregnancy; was considerably emaciated; pulse eighty-eight; thirst; dryness, and some heat of skin. Having another patient in labor, I left her, with directions to encourage the labor, by warm teas and a foot bath.

At 6 o'clock, I returned, and finding the same frequency and severity of pain, with only a very slight increase of dilatation, I first bled her about ten ounces, and followed it by morphine, in one-third grain doses every hour, until a grain had been taken. By 9 o'clock, the dilatation had made very decided progress. About half past 10, the waters escaped, yet it was not until after 11, that the head passed through the os-uteri, and not until a quarter past 12, that the child was born, in a state of nearly total asphyxia. It was resuscitated by the usual means, and is now a hearty boy, of some twenty pounds,

I commenced giving the chloroform, in moderate doses, about half past 9 o'clock. Her first exclamation, after breathing it was, "why did you not give it to me before!" and she then commenced asking for it, over and over again, after every pain, in the most earnest and plaintive manner; and so continued until the termination of the labor. The relief was very great; I never saw it more so, except total anæsthesia was produced. She cried out, more or less, with every pain, and occasionally, when I let a pain go over, without the chloroform, she would complain greatly.

At half past 11 o'clock, she had taken between four and five drachms, and I had only about half a drachm left, and wishing to keep this for the last two or three pains, I stopped giving it. The immediate increase of her suffering was apparent to all, aud she begged so hard for it, she got it all away from me, some time before the conclusion of the labor, which was finally terminated amid heavy suffering.

Just as I had the child safe, I heard a gush of blood from my patient. She was flooding. I found the afterbirth lying partly within, and partly without the mouth of the womb. It was immediately removed, and the contraction of the uterus secured. While I was pinning the binder which had been passed around her, a glance at her face showed her to be fainting, although there had not been any renewal of the flooding since the removal of the placenta. There had been only that one discharge. The removal of all pillows from under her head and shoulders, the dashing of a little cold water into her face, the opening of the windows and door, was but the work of a moment, and in a minute or two afterwards, she came to. I now noticed that the pulse had risen from 96 or 100, at which, it had been during the whole labor, to from 130, to 140. She continued faint for an hour; had a great desire for cold water, her thirst being intense; some brandy which I gave her, she did not like. Finally, some Wine of Ergot, I had sent for, being brought to me, I gave two teaspoonsful, under the idea, to secure by it a firm contraction of the uterus, as a preventive of further flooding. She had scarcely got it down, before she said she was sorry she had taken it, and in a few minutes more, vomited with great force and severity. She did not get faint again. I made no more efforts to give either the Ergot or brandy. Half an hour afterwards she took some tea, but this also brought on vomiting; so nothing more was given, except a spoonful of cold water, occasionally. I left her at 2 o'clock, quite comfortable. Her pulse had fallen to 110. I visited her late in the afternoon, and found her quite cheerful; no more vomiting; pulse 88. Directed some light chicken broth. It was prepared as soon as convenient to do so, and given her, but with the effect of renewing the vomiting. It was very distressing for a while, and her friends, in alarm, sent for me. I soon ascertained that there was no renewal of the flooding, as I at first feared; her pulse kept steadily at 88. I turned my attention wholly to quieting the stomach. A strong spice poultice was placed to the epigastrium, and all drink forbidden. Small pieces of ice were allowed to quench her thirst. She got to sleep after a while; and I left her after two or three hours, in a very comfortable condition. This was the end of my anxiety about this case. She convalesced without an unfavorable symptom, and without needing more than a light dose of medicine when the milk came.

I think I should have avoided the flooding in this case had I examined carefully and repeatedly whether the womb kept well contracted after the delivery of the child. I might have anticipated its possible relaxation, *first*, from her general exhausted state, and *second*, after such a severe labor; but my attention was so exclusively directed to the child that I neglected this precaution.

Had the Chloroform anything to do with this flooding? Was the asphyxia of the child caused by it? My own opinion is, that neither can be referred to it.

I would also add that there was not at any time more than a momentary loss of mind caused by the Chloroform; although at times she became somewhat indifferent; that is, less quick to notice what we said to her, or to do what was desired, than was natural with her. She afterwards said she did not remember much even of the conclusion of the labor, although her cries were then such as to shew she was acutely sensible. This want of memory was probably due more to the long continued faintness than to the Chloroform, which she had nearly ceased to take for three quarters of an hour before the conclusion of the labor.

*Case 8th.* Mrs. R. Was called the morning of January 2d, 1849. First labor. She had fallen and hurt herself the evening of December 30th, and had been in pain ever since. Says she has not slept for three nights, but she was soon awakened by pain in the back. She is thin in flesh; aged 21 years. I found general soreness of the abdomen upon pressure, pulse variable, from 100 to 110, and rather hard; lips very red and dry; cheeks flushed; skin hot; great thirst for cold water; no other kind of drink would satisfy her. On examination found the os-uteri dilated to a diameter of two inches; the head low down behind the pubes; membranes entire. She made a great complaint of her back; the pain seemed like a constant hard aching, instead being of the cutting kind. She desired greatly to go to sleep. Complained of bewilderment from want of sleep. Her bowels had been well moved the day before.

℞., v. s. ad ℥xii—℞., morph. gr. ¼ every hour until there is some relief.

At 11 o'clock found her easier, but still no sleep. She was sitting up; could hardly be persuaded to lie down. She had taken three of the powders of morphine. I directed the other quarter grain powder to be given in the afternoon.

7P. M. Found her in more pain, caused principally by distension of the bladder. I drew off a quart of water, with great relief. No progress made in the labor since morning. Membranes entire, and the head of the child, although low down, easily moveable, thus not affording any physical reason for the retention.

Symptoms generally as in the morning. Has not slept any during the day. Expressed a great desire for something to make her sleep. I thought I would give Chloroform for this purpose; so putting thirty or forty drops on the sponge, I gave it to her to breathe; as soon as I thought her asleep, I took away the sponge. She remained apparently asleep for five minutes. On awaking she immediately asked for more, and I tried the experiment over again. I then directed morphine as in the morning and left her.

Was sent for again at 5 P. M., Jan. 3rd., and found quite an accumulation of urine which required removal with the catheter. She had slept only during the early part of the night. Had been in constant, although not what we would call regular labor-pain, for some hours. Symptoms generally, precisely as yesterday. There was however considerable progress of dilatation. At 8 o'clock it was sufficiently advanced to rupture the membranes. There was no advance of the head in consequence of it. Sent for my forceps, and at 9 o'clock applied them; which was done with entire ease. Then pouring forty or fifty drops of Chloroform on the sponge, I directed her to breathe of it. She inhaled readily, and in less than half a minute was rendered sufficiently insensible to attempt extraction. As she had no regular pain, of course I had to deliver without it. This was very readily accomplished after two or three efforts, occupying not over two minutes. She did not come out from the state of anæsthesia until after the birth of the child, and she declared herself insensible at the time. The child was living and well.

There was here some restlessness at the time of extraction, a kind of an attempt to bear down, especially at the moment of the passage of the body through the vulva. The voluntary muscles of the abdomen were but moderately used; the womb contracted down well; there was no hemorrhage. After a few minutes, on examination, the placenta was found lying partly within, and partly without the womb, from which position it was readily taken. In less than five minutes after the woman was laid comfortably in bed, she was fast asleep, and so slept most of the day unless wakened. Her pulse and feverish symptoms generally kept up the most part of two days, when they all subsided and she rapidly convalesced.

*Case 9th.* Mrs. O'B. *January 4th.* Called at 10 P. M. Found her in hard labor with her third child. With the first she was in labor five days, and was finally delivered with the forceps. With the second, after suffering an entire night with no prospect of immediate delivery unless assisted, she asked for the forceps, and seeing no objection to their use I applied them safely both to herself and to the child.

This time the os-uteri became fully dilated by midnight, when the waters were discharged. I was pained to find a prolapsus of the cord. I tried for some time to pass it back above the head, but the amount down was so large that I could not keep it there; and finding, after a while, the pulsations to be getting feebler and feebler, I determined to apply the forceps, although the head had as yet only fairly engaged in the superior strait. I had no difficulty in applying the instruments and in locking them, but on making traction I found the anterior wall of the vagina, and I thought the bladder, also, coming down before the head. It was a complication that had never before occurred to me, and I am yet at a loss for the true reason of it. After many fruitless attempts to support the prolapsed membrane in its place until I could bring the head below it, I reluctantly gave it up and removed my instruments. The head of the child was too large to allow of any such manœuvre.

It is not often that we see more regular and better pains than this patient had for the next four hours. They were forcible, expulsive, came on at short intervals, and left her *clear* as it is called. It was during a good portion of this time that I gave the chloroform. She breathed it well, but did not express that satisfaction from its use, I had generally remarked. On being asked, she said it relieved her, and two or three times she called for it. The head came down very slowly, and at 4 o'clock, had not accomplished more than three-fourths of the distance through the cavity of the pelvis. It had passed low enough, however, to get below the fold of the vagina, which had prevented my using the forceps. I did not see that this fold at all interfered with the regular, slow, progress of the labour. It gradually disappeared as the head descended. The cord had long ceased to pulsate.

I now determined to try the forceps again. Their application, the use of the chloroform, and the delivery were so precisely similar to the case just above related, that a description would be but a repetition. The delivery of the child was painless to her. It was still-born. She convalesced rapidly, having no trouble, except for two or three days with her milk.

*Case* 10.—Mrs. H. A small short woman with her first child. I first saw her on the evening of January 14. She had been in pain most of the day. I found the os-uteri dilated only to the size of a dime ; pains quite inefficient; pulse 100; feverish symptoms. Prescribed morphine gr. $\frac{1}{4}$., to be repeated once in a couple of hours, if necessary.

*January* 15, 10 *o'clock.* She had obtained only moderate relief from the morphine. Still feverish and thirsty. ℞., v. s. ad ℥ xvi. Was called at 3 o'clock, in the afternoon. Found her in good pain, and dilatation making progress. Gave her chloroform from a quarter past 4, to a quarter past 5 o'clock, on the accession of nearly every pain, with the usual relief. She was also somewhat indifferent to it, but not to the degree shewn by the last patient. At a quarter past 5, the waters escaped, and on making an examination, I discovered the face presenting, the chin pointing to the spine of the right ischium, while the forehead was behind the ramus of the left pubes. Os-uteri not enough dilated to slip over the head, nor did it become so until near nine o'clock. During this time I gave her Chloroform at intervals, at her request, although I did not encourage her in taking it after I discovered the real position, fearing something might not go right, and then it would very likely be laid to the Chloroform. The case proved a very interesting one, but not coming within the scope of this article further than in its connection with the use of Chloroform, I do not give the particulars. I will only add, that finally, at 10 o'clock, I applied the forceps across the face (Dr. B. Burwell in consultation,) and safely delivered my patient of a living child. The only mark of the forceps upon its head was an excoriation of the cuticle behind its left ear, about the size of a half dollar piece. It readily healed. Its left eye was much swollen and ecchymosed, this being the lowest presenting point. Both mother and child did well.

*Case 11th.* Mrs. S. In labor, January 21st, with her fourth child. I saw her at 2 o'clock P. M. Everything favorable. Child born at 4 o'clock. Her pains being pretty severe and rapid, I proposed, at half past 3, that she should take the Chloroform, to which she consented. Her exclamations after the first two times she breathed it, were, that it made her pains sharper. This was because she commenced breathing it just at the commencement of the pains, and she did not get under its influence before they became quite sharp; but by having her breathe it in the intervals, she soon experienced its soothing effects. I never had a patient shew more avidity for it. It was sometimes rather amusing to see how she clung to the sponge when I directed its removal from her face. Although so much comforted by it, she did not get enough to become once insensible. She breathed it just half an hour on each return of pain. It did not interfere, in the least, with the full action of the voluntary muscles, nor did it alter the natural beat of her pulse. Child active. Both did well.

*Case 12th.* Mrs. W. January 23rd. Had been in pain all night. The waters escaped at ¼ to 1. P. M. Os-uteri fully dilated, position 1st of the vertex. First child. General condition favorable. Pains severe and expulsive. She commenced breathing Chloroform at ¼ past 1 o'clock and continued to do so at the commencement of every pain for an hour. I then let her have four or five pains without it, occupying about fifteen minutes; then commenced again, and continued it every pain for twenty minutes until the child was born. She was not made insensible; at least she declared she did not once loose herself; but I thought differently two or three times. There was no delirium. She evidently did sleep in the intervals of many of the pains.

The pains came on so suddenly, that I found myself obliged to give the remedy in the intervals, just before they might be expected. If one was allowed to come on before she breathed the chloroform, I had to desist from giving it, for that time; for they were of so expulsive a nature, that she could not breathe more than a short catching of the breath.

Shortly after the birth of the child, she asked what time it was, and expressed her surprise that it was so late. The two hours I had been with her, did not seem to her to have been one half an hour. This shews that she must have been so far affected by the remedy, as not to be fully sensible of the true lapse of time. She did not loose the free use of the voluntary muscles. There was no hemorrhage; the after-birth came away at the proper time, and both herself and the child did well.

*Case* 13*th*, Mrs. M.—Was called at 3 o'clock A. M., Feb. 18th. She was in labor with her sixth child. Her health had not been very firm, and I found her quite low spirited. She had never felt so depressed at any previous labor. It did not seem to her that she could live through the pain necessary to give birth to her child, and she was determined, if possible, to get relief by means of chloroform. It was for this purpose, that she had chosen me to attend her. "Now, Doctor, give me just all you dare to," was the direction I got from her, when about to commence its inhalation. This was at 4 o'clock. The os-uteri had reached nearly its full dilatation, and the pains were getting quite sharp. She was impatient of them; and while they were on, she was in constant motion, rolling from side to side, drawing up and extending her limbs, so it was quite inconvenient to wait upon her. She said she had not been so, in previous labors, and I could only account for it, by her loss of self control, and her general feeling of self abandonment, regardless of consequences.

I expected to control this restlessness by the chloroform, but was disappointed in it; although, for about half an hour, there was nearly total anæsthesia. When I noticed this effect from its use, I desisted giving more, or making attempts to give enough to stop the restlessness. I knew she did not suffer from pain, and this result was all I sought to obtain. The uneasiness lasted only while the pain was on. She cried out, as though suffering, and those unaccustomed to the use and effects of chloroform, would have been confident she did suffer. How easy, without due caution and experience, to have pushed the remedy, and to have done irreparable injury. Of course, there was all want of concert of action between the voluntary and involuntary muscles. Had there been this concert, I think the child would have been born some eight or ten minutes sooner. The chloroform did not seem to have any effect in preventing this concert. It was equally wanting before she commenced its inhalation. It was curious to notice, towards the close of the labor, with what force the head was thrown downwards, during each contraction.

The child was born at 5 o'clock. She was therefore one hour under the influence of the remedy. She remembered the most that occurred the first half hour; nothing during the remainder of the labor, except, indistinctly, the passage of the child into the world. As in the twelfth case, the time I had been with her seemed much less than it really had been. Her exclamations after first breathing it were, "How it relieves me; how clear this pain has gone off; I am perfectly easy." Ten minutes after the completion of the labor, I overheard her telling a friend, that she no more felt any effects from the chloroform, than if she had not taken any.

I gave it in doses of only twenty or thirty drops; and only on the accession of each pain. One wetting of the sponge generally did for two pains, and sometimes for three. The pains came in rapid succession, every two or three minutes. It is worthy of notice, how a small quantity, frequently repeated, will produce total anæsthesia.

Her pulse and respiration were unaffected by it. I was careful to see that she breathed deeply and freely during the intervals of pain.

Her getting up was excellent. The child did well.

*Case* 14*th*. Mrs. R.—Taken in labor with her second child, March 1. She was equally impatient of pain, with the last case, but had but little of that restlessness. When in pain, she would help herself what she could. Every thing going on favorably, at her request, half an hour before the close of the labor, I gave her chloroform. It was continued on the return of each pain, until the child was born. Total anæsthesia was not produced; and as usual, when it is not, she made considerable outcry during pain, yet calling for more, and declaring the relief she obtained. After the second inhalation, she had quite a fit of laughing, which, however, was but momentary. She did well. The child hearty.

*Case* 15*th*. Mrs. M.—Was called at 6 o'clock A. M., March 10. She had been in pain since 1 o'clock. She was twenty-two years of age; of short stature; the heaviest weight she ever attained was ninety-six pounds. Says she is in constant pain; but once in ten minutes there is an exacerbation of pain, across from the pubes to the back. Is quite thirsty; has a dry skin and parched lips. Pulse 84; moderately full. There being no change of symptoms, at 8 o'clock, and finding the os-uteri high up, thick, and open just sufficiently to admit the end of the finger, I bled her about sixteen ounces. This produced a decided effect, after a couple of hours, in reducing the fever, and she did not again become so thirsty. At 11 o'clock her pains began to get quicker and more severe, but of the same aching character as in the morning, and never left entirely clear. Quarter past 2 o'clock P. M.—Still an increase in the severity and frequency of the pains. Commenced the administration of the chloroform, in doses of about thirty drops, and repeating the inhalation every pain. This amount generally lasted for two, and sometimes three pains.

**3** *o'clock.* Os-uteri dilated to two inches in diameter. Pulse variable from 88 to 100. Pains evidently quite severe. The inhalations were continued at her especial desire. A part of the time she held and used the sponge herself, and breathed from it with avidity, yet with discretion. The Chloroform made her talkative, yet did not destroy all sensation. It made the pains "tolerable" as she expressed it. Its general effects were pleasant, and she desired it every pain. Sometimes it required only three or four inhalations, sometimes six or eight, according as the sponge was "strong" to use her words; that is, as it had, or had not recently been wet with the Chloroform.

*At* ¼ *to* 5 the os-uteri had become nearly fully dilated. Having used all the Chloroform I had with me, she went without some twenty minutes while I procured more. This was the longest interval that its use was dispensed with.

*At* 5½ *o'clock* I ruptured the membranes, the head of the child still high up. It did not descend and pass through the os-uteri for three-fourths of an hour afterwards. Her pains were now some five minutes apart; they did not last long, nor were they very expulsive. Until 5½ o'clock I had used the Chloroform quite freely. Eight fluid drachms had been consumed, or over two drachms an hour. From this time until the conclusion of the labor, at 7½ o'clock, only two drachms were used; less than half the quantity that had been given before. The reasons for this were, 1st, the quantity she had already consumed; 2d, her pulse had never risen permanently to 100; 3d, I did not know but it might lessen the expulsive character of her pains.

The perineum yielded slowly and painfully. The after-birth was thrown off in ten minutes after the birth of the child. At 8½ o'clock her pulse had fallen to 88, and I left her comfortable.

She was at all times anxious to take the Chloroform; and especially did she complain during the last two hours that I did not give it more freely and frequently; the little I did allow her, she said, was only an agravation.

Only twice during the first three hours did she loose her senses for an instant or two; and also two or three times during the last two hours.

She felt sensibly the diminution of quantity in an increase of the pain. Her cries were greater; she was much more restless; could not lie still during the pains, nor make any steady effort to help herself in the way of bearing down. She frequently said she was sensible of everything; spoke of the acuteness of her pain; how the Chloroform, when I gave it to her, relieved her, and made the pain tolerable; and was constantly asking for more, and telling me to give her a great deal. It really required some firmness to deny it to her.

I would also add, that twice during the last hour there was a little discharge of blood from her nose, amounting altogether to a few drops only, and caused probably from the irritation of the schneiderian membrane by the vapour of the Chloroform, and her frequent violent efforts in snuffing it into her nostrils.

I would add, as a fact of some importance, that her breath smelt decidedly of the Chloroform at 8½ o'clock, one hour after the birth of the child.

*March* 11*th*, 9 A. M. Pulse 76; slept three or fours during the night; has some thirst; looks well, and is cheerful. Had considerable aching through the pelvis all night, but which was greatly relieved after urination.

On enquiry, I find she does not remember much about the last hour of her sickness, except the general distress she felt, and her joy when she heard the child cry; nor does she remember much that she said during this time, or that was said and done by her friends.

She convalesced finely. The child cried lustily the moment of birth. There was no symptom of asphyxia, although its mother's system had been so saturated with the Chloroform. It also did well.

This patient took more Chloroform than I had before, or have since given to one person. The case is suggestive of the following reflections:

*First.* The consumption of ten drachms of Chloroform in 5¼ hours had no effect in causing inflammatory action, although it might be supposed that her system was disposed to take it on, as indicated by the thirst, heat of skin, pulse and the undilatability of the os-uteri before bleeding, in spite of constant pain.

*Second.* As to the quantity necessary to procure relief. About seven drachms were given during the first two and and a half hours. This was might, and could it have been used without waste, one half this quantity possibly, have sufficed just as well. One of the attendants remarked between four and five o'clock, that she had never seen any labor so easy and quiet.

When an ounce had been used, I thought that it would be better to let her suffer some than to continue its use so freely; and, as a matter of course, she was afterwards more sensible to pain.

*Third.* As to particular effects. It caused talkativeness. She knew well enough what she said, but there was the disposition to talk which she did not care to control. The first smile seen on her face for some hours was about fifteen minutes after commencing the inhalations. I do not think it had any effect in promoting the dilatability of the os-uteri, or of the perineum, nor did it increase the lubrication of the passages. It has been the fairest case I have had, in which to expect such a result.

*Fourth.* I think the remedy is cumulative as it is called; at least it shewed itself to be so in this case. The first time I gave it to her, she declared it did not help her at all; it only made her head light. From, and after the fourth trial, she acknowledged the relief obtained from it.

*Case* 16*th.* Mrs. M. Was called in the evening March 12th. She was in labor with her fourth child. Every thing going on favorably. I gave the Chloroform only during the last fifteen minutes. She said she felt strength, as well as ease from its use. Both herself and child did well.

*Case* 17*th.* Mrs. W. Confined with her first child March 14th. She was a stout, hearty woman, and in excellent health. She had been in pain most of the night. By 10 o'clock the pain had become frequent; and as they became more severe towards noon, she lost her usual liveliness of spirits, and lay in a dull, apathetic condition; sensible enough to pain, but indisposed to move, even to fix the pillow under her head; she refused drink; said little or nothing; it was even difficult sometimes to get her to answer a question, except by a monosyllable. When in pain she would exert herself pretty well, occasionally making some remark expressive of her suffering, and of her expectation of not living to get through with it.

I offered her Chloroform as a means of relief. She breathed it for a few pains, but made no remark as to the amount of ease obtained; and not seeing much change in her actions, I doubted whether she did get much relief, and so discontinued it. This was about 12 o'clock. After half an hour her suffering evidently becoming greater, and desirous of knowing whether she had experienced any relief, I asked her about it, and whether she wished more, saying distinctly, that I did not wish to give it unless she felt decidedly relieved by it.

She expressed her sense of relief while she breathed of it, and her willingness to take more. I therefore gave it at intervals until the child was born, which was about half past 1 o'clock.

Still, she did not at any time come out from that apathetic condition, nor become lively and in better spirits as was seen in the fifteenth case; she did not ask for it; nor tell when the pain was coming on. In fact I did not experience my usual satisfaction from giving it. Both mother and child did excellently well.

When a patient lies in such a condition, it is difficult to judge of the effect of the remedy. You have only one symptom by which to judge of the amount of relief obtained; the expressions of suffering, when the pain is on.

I want an active intelligence, when I administer chloroform, in labor; one that I can depend upon, to express any disagreeable symptoms or sensations from its use, as well as the amount of relief obtained. If they do not give utterance to the comfort they derive from it, as they are generally so prompt to do, I do not feel certain that they will inform me of any uncomfortable feelings that may be produced by it. For these reasons, I determined, after the experience of this case, not to continue the remedy in similar ones, two or three of which have occurred to me. None of them are included in my list of cases.

*Case* 18*th.*—Mrs. T., *March* 17*th.* This was a rapid case, with sharp pains. Third child. I had an opportunity of giving it only for about ten minutes. After the first inhalation, she exclaimed, "how bad it makes my head feel." On asking her to particularize the sensation, she said it was a sense of dizziness and vertigo, with nausea at the stomach. Having experienced these sensations myself, from its use, I did not feel that they afforded a sufficient reason for its discontinuance. They all passed off with the second inhalation, and she expressed her satisfaction with the relief obtained. Both mother and child did well.

*Case* 19*th.*—Mrs. G. In labor with her first child, March 17th. On visiting her at 8 o'clock, P. M., I found that she had been under the care of another physician, for about thirty-six hours, but from some disagreement between them, he had left her. Her pulse was 140; skin dry; thirst; countenance and voice firm; pains frequent, but unequal and inefficient. On examination, I found that the waters had escaped; the head was low down; os-uteri I at first thought to be fully dilated, but a pain coming on, I felt it tighten before the head, and to become about two and a half inches in diameter; there was an unnatural warmth about the passages. The woman was young, of the middling size, and had generally enjoyed good health. I bled her to ℥ xvi, and gave morphine, one-third of a grain, every hour, until some relief was obtained. In two hours, she had got quite comfortable; the pains had nearly ceased; pulse had fallen to 112; disposition to sleep.

She told me that she had been feverish all day. The blood I had drawn, was entirely free from the buffy coat. I saw a vial of Tinct. Ergot, on the table, of which she said she had taken a number of doses during the afternoon.

I now left her for the night, and did not see her again until the next morning, at 9 o'clock. She had rested tolerably well, until about 2 o'clock; had had good pains since 4 o'clock. I found the os-uteri fully dilated, and the head almost down to the perineum. Her pulse had risen to 130. An hour now passed, during which I paid every attention to facilitating the labor; the pains were severe, and she bore down well, but no progress was made. I now (10 o'clock) deemed it expedient to apply the forceps. She was placed in the proper position, and the instruments applied with the greatest facility. Chloroform was administered in the full dose, and the child delivered while she was insensible. This anæsthesia did not last over half a minute after the birth of the child.

Its head was moderately hydrocephalic; a large spina-bifida existed at the lower dorsal vertebræ, and both feet were badly clubbed. It made a few gasping efforts at breathing, for some minutes, and then died. The afterbirth came away well. There was no flooding.

This patient's pulse kept up to 130, for two days, giving me serious uneasiness, but happily they fell when the milk came; she rapidly convalesced, and was sitting up by the close of the week.

*Case 20th.*—Mrs. W., *March 26th.* This patient had been unfortunate with her children, this being the first time she had reached the full term of gestation. Three times, within the last four years, had she miscarried. She is of a decidedly nervous temperament, of small stature, and general health only tolerably good.

She was in pain most of the night of the 25th, and by noon of the 26th, it had got to be severe. But when I first saw her, (at 1 o'clock) her greatest distress was in her head. The pains of labor, which recurred every eight or ten minutes, were almost unnoticed, in comparison. "Oh! my head, my head, it will split open," was her frequent exclamation. She was exceedingly restless. Her head was quite warm; extremities cold. She complained that she could not see well. Every thing had looked dark to her for half an hour, and once, shortly before I arrived, she could not distinguish her friends, one from another, when only ten feet from her. Her face was pale; her eyes had a very unnatural expression; pulse 96. I bled her about ℥ xvi, and gave an anodyne. She threw it up; I repeated it, and she threw it up again; and from this time, until the close of the labor, she had a turn of vomiting every few minutes; or rather, it soon got to be a gulping, or spitting up of such liquid as might be upon her stomach. It made no matter whether she took anything or not; the symptom was the same.

I have rarely seen a greater change, in the same time, in any patient's appearance, than she presented, in an hour and a half after the bleeding. The headache, although yet present, was greatly relieved; her face and eyes looked natural; her extremeties had become warm; pulse 84. Her pains were increasing in severity, and dilatation was making tolerably good progress.

In another half hour, her cries from pain becoming quite loud, I offered chloroform, as a means of relief. She breathed it, in the way and dose I am in the habit of administering it, with great satisfaction. It had no unpleasant effect upon her head; she said it relieved, rather than aggravated her headache. Such was the quietness with which she bore her pains, that the nurse (who was opposed to my giving it to her) seriously asked me, whether I believed her to be making as rapid progress as before. The birth of the child, a few pains afterwards, without a cry of distress, gave the best answer to the question.

It was about three quarters of an hour, from the time she commenced breathing the chloroform, until the birth of the child, She had an excellent recovery; the child did well. She told me the next day, that the best thing I did for her, was in giving her the chloroform.

*Case* 21*st*. Mrs. L.—This proved to be a case of twins. They were born, March 28th. The first was a presentation of the breech and feet; the second, of the head.

Few suffered more than she did, for nine days before labor, from pain and distension. A grain of morphine was taken (in one-third grain doses) nearly every night, with but slight relief. I bled her on the evening of March 26th, and afterwards gave the usual amount of morphine. She was able to lie down, that night, and slept for about two hours, the only time, for the nine days and nights. Her nights were spent in a rocking-chair, in which such sleep as she got, was very uneasy and unsatisfactory.

The waters escaped on the 26th, and it was not until the morning of the 28th, that she got regular labor pains. Her pulse was permanently at 120, all day, although she had but moderate thirst or heat of skin.

The first child was born at 12 o'clock, (noon) only by my drawing down the feet, and delivering. The pains, of themselves, were entirely inefficient to effect the expulsion of the child. In half an hour afterwards, pain came on. In another half hour, I ruptured the membranes of the second child. The head was soon forced low down in the cavity of the pelvis. Her pains, such as they were, recurred at regular intervals of five minutes, for the next three-fourths of an hour; when finding not the slightest advance, I applied the forceps, and delivered. Both children were living, and did well.

I gave chloroform in this case, only at intervals, and generally, at her request. After breathing enough of it, to feel its effects in easing pain, she would invariably fall into a deep sleep, sometimes, with snoring. This was not, as I thought, from the direct effect of the chloroform; but, on the contrary, such was her exhaustion from so much pain, and the consequent want of all good sleep for so many nights, that I think she would have fallen asleep immediately, upon obtaining relief, by whatever agency that relief might have been gotten.

Two or three times this sleep lasted for twelve or fifteen minutes, during which time, there would be an entire cessation of pain, although just before, the pain had recurred every four or five minutes. It was when they came on so often, that she would ask for chloroform. Such being the effect of the remedy, of course, any constant use of it was out of the question. It was not given during the delivery of the second child, solely because a pain came on, the moment I had the forceps locked, and I took advantage of it, to deliver, knowing that one pain would be sufficient. She had asked for it, and I had directed its administration, but the child was delivered before she had time to breathe of it.

As in the *eighth* case, this patient fell into a sound sleep soon after she had got placed comfortably in bed; and so sound was it, that I gave direction to have her gently roused, every few minutes. In fact, when I saw her at 8 o'clock, it was so sound, that I almost felt apprehensive of apoplexy. It was not until after midnight, that this apoplectic sleep ceased, and she became more wakeful.

Her recovery was slow. The symptoms, the most unpleasant, for the next two days, were a pulse constantly at 120, with a more than ordinary size, and great tenderness of the uterine tumor, giving me a constant fear of hysteritis. Luckily, on the third day, the pulse began to fall, and the tumor to subside, without a resort to any depletion, or other than a soothing, anodyne treatment, which it would be out of place, here, particularly to specify. From this time her convalescence was daily progressive.

*Case 22d.*—Mrs. W. Confined, April 7th, with her third child. The labor proceeding in every way satisfactorily, I administered chloroform during the last half hour, but not to the extent of producing entire anæsthesia. So far from it, I did not give it so freely as I might have done, to have given even more perfect relief, without taking away her intelligence. Both mother and child did well.

*Case 23d.*—Mrs. J. In labor, May 8th, with her first child. Her general health has been poor for some years; does not consider herself of a strong constitution. She lay in pain all night of the 7th of May. The waters escaped at 1 o'clock, P. M., of the 8th, before full dilatation. Her pain becoming quite severe, at 4 o'clock, I commenced the use of chloroform. She breathed it well, and it did not excite the least uncomfortable sensation; on the contrary, she said she felt it go right to her back, and numb the pain there. She was very enthusiastic in its favor, and asked for it, again and again. I at first continued its use for about an hour. Dilatation proceeded slowly. I left it off half an hour, and then returned to its use, with the same relief as before.

By this time, the head of the child had passed through the os-uteri, and had nearly reached the perineum, where it stopped farther progress. It did not come down sufficiently, to make any pressure upon the perineum. Thinking, that, by possibility, the chloroform, while it eased the pains, might really lessen their expulsive force, I again discontinued its use. No more progress was made, than before, although an hour passed by, with as good pains, and as forcible efforts, on her part, to assist them, as is ever seen. After another half hour, she ceased to help herself, declaring her inability to do so longer, and asked for relief. No chloroform was given during this hour and a half.

I sent for my forceps, and applied them. On the first pain, I was enabled to bring the head against the perineum. But I did not dare to hurry its exit through the vulva. There was no dilatation sufficient to permit it. However, twenty or twenty-five minutes of pain prepared the way, and removing the instruments, which, until now, had been left on, but used only to a moderate extent, the child was born; a hearty, lusty boy. Chloroform was not given during the application of the instruments and delivery of the child. She convalesced rather slowly, but without sickness.

This concludes the cases I proposed to give, and with two or three exceptions, includes *all the cases* in which I have administered, or attempted to administer chloroform in labor.

These exceptions are, *first;* two or three cases in which I commenced its use, but almost immediately discontinued it, without having brought them, only momentarily, if at all, under its influence. It was discontinued solely because they did not breathe of it, to suit me, and not because there was the least unpleasant symptom produced.

*Second.*—One case where the woman attempted, three or four times, to breathe of it, but could not. There was something in the smell of it, that "stifled" her, as she called it. As soon as it was placed near her face, she was unable even to draw in her breath. She is a very intelligent person, and was anxious to try it, but positively could not bear it near her.

*Third.*—I gave it during one pain, to a woman, to whom I was called in consultation, by Dr. H. D. Garvin, on the 3d of January last. She had been in labor all one night, trying to get along without a physician. Dr. Garvin was called, and finding, after a while, there was no progress to the labor, although the pains were apparently good, he sent for me to bring my instruments. I found her in great pain, very tympanitic, and very tender and tense across her bowels; pulse 140; extremeties cool; face blue, and in a profuse sweat. It occurred to me, to try what effect chloroform would have in quieting her. I put thirty or forty drops of it on the sponge, and told her to breathe of it. As soon as she got under the influence of it, she became flighty, without being calmed by it. This lasted but for a moment, however, and the remedy was not again given. I proceeded to apply the forceps, and after a good deal of trouble, delivered her.

We did not dare to bleed her, although there were decided symptoms of peritonitis. Anodynes and warm drinks were given. She sank rapidly, and died the next day. No post-mortem examination could be had.

I had intended to close this article with a general summary of my experience with the remedy; the cautions necessary to be observed in its administration, and the indications and contra-indications, as furnished by different symptoms, to its use or continuance; but I find myself unable, at present, to carry out this intention, and must defer it until the next number.

BUFFALO, May 19th, 1849.

